# P04.81. Mapping the natural health landscape: New Zealand-based CAM professionals survey

**DOI:** 10.1186/1472-6882-12-S1-P351

**Published:** 2012-06-12

**Authors:** R Vempati, J Dunn, P Cottingham, D Sibbritt, J Adams

**Affiliations:** 1 Wellpark College of Natural Therapies, Auckland, New Zealand; 2 New Zealand College of Massage, Auckland, New Zealand; 3 University of Newcastle, Newcastle, Australia; 4 University of Technology, Sydney, Australia

## Purpose

Complementary and Alternative Medicine (CAM) is increasing in New Zealand (NZ). As public interest in the use of CAM grows, political recognition has become increasingly topical. However, no NZ-based, empirical study of CAM professionals has been performed. The survey reported here provides a direct response to this important research gap. It examines key aspects of practice, demographics, attitudes and beliefs held about integrative medicine, CAM regulation and research attempting to quantify CAM contribution towards public health in NZ.

## Methods

An online survey examined key aspects of practice, demographics, attitudes and beliefs held about integrative medicine, CAM regulation and CAM research and quantified CAM contribution towards public health in NZ. CAM practitioners were contacted via their professional bodies and individual associations. Participation response rates for individual CAM professions and CAM as a whole were calculated.

## Results

200 practitioners have responded. The majority of CAM professionals are self-employed females, aged 45-54 years. Main modalities practiced are: herbal medicine, homeopathy, naturopathy, nutrition and massage with many CAM practitioners (40%) practicing multiple modalities. The majority believe they should be integrated into main stream health care with most referring to GPs at least 1-5 times/year (49%) and vice versa (39%). Statutory regulation was seen as essential by 68% of CAM practitioners with 90% supporting registration either statutory or voluntary.

## Conclusion

There is a high level of integration, in the form of referrals between primary health care physicians and CAM practitioners. Statutory CAM regulation is a pressing need to integrate with the mainstream health sector. Qualified CAM practitioners have a better understanding of research findings although they have limited ability to conduct research. The development of research capacity including CAM research funding is essential in NZ, hence local and national health care authorities need to recognize and support CAM contributions to NZ health care.

